# Homoparenting as a public health issue: a scoping review

**DOI:** 10.11606/s1518-8787.2023057005447

**Published:** 2023-10-24

**Authors:** Romeu Gomes, Tereza Setsuko Toma, Jessica De Lucca Da Silva, Fernando Meirinho Domene, Adriano da Silva

**Affiliations:** I Fundação Oswaldo Cruz Instituto Nacional de Saúde da Mulher, da Criança e do Adolescente Fernandes Figueira Departamento de Ensino Rio de Janeiro RJ Brazil Fundação Oswaldo Cruz . Instituto Nacional de Saúde da Mulher, da Criança e do Adolescente Fernandes Figueira . Departamento de Ensino . Rio de Janeiro , RJ , Brazil; II Hospital Sírio-Libanês Diretoria de Compromisso Social São Paulo SP Brazil Hospital Sírio-Libanês . Diretoria de Compromisso Social . São Paulo , SP , Brazil .; III Secretaria de Estado da Saúde de São Paulo Instituto de Saúde Núcleo de Evidências São Paulo SP Brazil Secretaria de Estado da Saúde de São Paulo . Instituto de Saúde (NEv-IS). Núcleo de Evidências . São Paulo , SP , Brazil; IV Fundação Oswaldo Cruz Escola Nacional de Saúde Pública Sergio Arouca Departamento de Estudos sobre Violência e Saúde Jorge Careli Rio de Janeiro RJ Brazil Fundação Oswaldo Cruz . Escola Nacional de Saúde Pública Sergio Arouca . Departamento de Estudos sobre Violência e Saúde Jorge Careli . Rio de Janeiro , RJ , Brazil

**Keywords:** Homosexuality, Family, Sexual and Gender Minorities, Health, Review

## Abstract

**OBJECTIVE:**

To map global scientific production on homoparenting in the field of collective health or public health.

**METHODS:**

In terms of methodological procedures, a scoping review was carried out, guided by the following question: What are the aspects addressed in global scientific production regarding homoparental families in the field of collective or public health? The searches were carried out in seven sources of scientific literature, including 58 studies, involving scientific articles and dissertations. The analytical treatment given to the studies, most of which were qualitative, followed the content analysis technique in the thematic modality.

**RESULTS:**

The results indicate that the perceptions of homosexuals and professionals about the care provided and health services in general was the topic addressed by the largest number of studies (n = 31), followed by heteronormative context of health services (n = 26); disclosure of sexual orientation (n = 20); fertilization (n = 14); educational information and actions (n = 5).

**CONCLUSION:**

Although the issue of same-sex parenthood has been discussed in some health sectors, there is awareness that it is necessary to rely on a consolidated basis through numerous studies when discussing this issue. It is concluded that, among other aspects, the scope of this review is not sufficiently problematized within the scope of health professionals’ training and performance.

## INTRODUCTION

The family has been one of the central focuses in several instances of public health. As an example of this, the Family Health Strategy stands out, one of the models for organizing services in the Brazilian Unified Health System (SUS). In this and other instances, the commonly used family reference is the traditional model, which originates from the union between a cis man and a cis woman. This union establishes, in the contexts, texts and relationships of the health area in general, the hegemony of heterosexual parenting, disregarding homoparenting or same-sex parenting, which is the theme of this article.

In order to discuss same-sex parenting, it is necessary—based on anthropological studies—to take into account that the types of relationships considered as family can be seen in different ways within their own societies, not being limited to genealogically defined relationships ^1^ . Considering the different types of relationships, gay and lesbian families can include lovers, co-parenting, adopted children, children from a previous relationship, and children conceived through alternative insemination ^1^ .

Although the issue of same-sex parenting has been discussed in some health sectors, the need to have a consolidated base with numerous studies when problematizing this issue is well-known. One of the dimensions to be covered is the formation of an analytical framework, considering the specialized literature, that can serve as a reference for incorporating the discussion about the object of study both in the logics and in the scenarios of collective health practices.

Zambrano ^2^ notes that:

Homo-parenthood is a neologism, created in 1997 by the Association of parents and future gay and lesbian parents (APGL), in Paris, nominating a situation in which at least one adult refers to themselves as homosexual who is or wishes to be a father or mother of at least one child. (p. 127; our translation) ^2^ .

Ribeiro et al. ^3^ (p. 3592), based on Zambrano ^2^ , observe that homo-parenthood is constituted from at least four situations:

[…] by children born in a previous heterosexual relationship, by legal or informal adoption, by the use of new reproductive technologies that enable the birth of biological children, and by coparenting, in which care for the child is exercised in a joint and egalitarian way by partners ^3^ (p. 3592; our translation).

With the aim of placing same-sex parenting in the context of the changes that have been taking place in the family institution, it is observed that the patriarchal family has been questioned since the end of the last millennium. In recent years, the dissociation between heterosexuality, patriarchy and reproduction of the species reinforced the gay and lesbian movement’s struggle to have legal recognition of getting married, starting a family, and having children ^4^ .

In line with this claim, the exclusivity of having a cis man and a cis woman to form what is called a family is questioned, so that if the bond of affection is considered central to the family institution, the union between people of the same sex can be considered as family ^5^ .

Although the discussion of the subject is not new, it is inferred that—in the health area in general in Brazil—publications on same-sex parenting are scarce. A concise survey, carried out on July 7, 2021, with the expressions “homoparentalidade *AND* saúde,” found only two articles in the Scientific Electronic Library Online (SciELO) and four in the *Portal Regional da Biblioteca Virtual em Saúde* (BVS).

Clearly, these quick surveys do not represent the state of the art of the subject within the scope of Brazilian scientific production, requiring more in-depth searches, in a systematic way, with a wide range of databases.

In this sense, a scoping review is proposed to be carried out, with the aim of mapping global scientific production on homo-parenthood in the field of collective health or public health.

In Brazil and in some Latin American countries, there is a difference between collective health and public health. The former, according to Paim ^6^ , refers to a field integrated by knowledge, practice, and ideology, differentiating itself from both public health and the hegemonic medical model and articulating science and practices for the formulation and conduct of consequential policies. Thus, the collective is not just an abstract population or population segment, and actions aimed at the collective are not exclusive to the State. In the international panorama, in general, the term collective health does not appear, but rather public health, which encompasses measures designed and adopted mainly by the State to ensure the population’s physical, mental, and social well-being. In this sense, the scope of this review is analyzed in the realm of collective health or public health so that production is not reduced to the Latin American sphere.

## METHODS

We carried out a scoping review based on the methodological framework of the Joanna Briggs Institute ^7^ . For the reporting of this review, the recommendations of the PRISMA Extension for Scoping Reviews ^8^ tool were used. A research protocol has been registered in the Open Science Framework (OSF) ^9^ .

### RESearch Question

The question “What are the aspects addressed in global scientific production regarding homoparental families in the field of collective or public health?” was constructed with the help of the acronym PCC (Population: cisgender homoparental families; Concept: global scientific production; Context: collective or public health). We decided to work with an open and broad question to obtain a greater diversity of scientific production on the subject.

### Inclusion and Exclusion Criteria

The inclusion criteria were primary and secondary studies, including documents, reports, dissertations, or theses, available in English, Portuguese or Spanish, which addressed issues related to policies, health programs and access to services for cisgender homoparental families in the context of public health or public.

Studies that referred to contexts other than collective health, that analyzed configurations of non-cisgender same-sex families, or that were in languages other than those mentioned above, were excluded.

### Data Sources and Search Strategies

The construction of strategies and the searches were carried out by a librarian in the following data sources: PubMed/MEDLINE, *Literatura Latino-Americana e do Caribe em Ciências da Saúde* in the Virtual Health Library (VHL/LILACS), SciELO, Scopus, Web of Science, Dimensions (July 2022), and *Biblioteca Digital Brasileira de Teses e Dissertações* (BDTD) (September 2022). Based on the combination of keywords structured from the acronym PCC, the MeSH terms (Medical Subject Headings) were used in PubMed and DeCS (Health Sciences Descriptors) in the VHL, adapting them to the other databases. The search strategies with the keywords used in each database are available in the protocol of this review registered in OSF ^9^ .

### Study Selection

The studies retrieved from the information sources went through a selection process based on pre-defined inclusion and exclusion criteria. After excluding duplicates, two reviewers independently carried out the screening based on reading titles and abstracts, using the bibliographic manager Rayyan QCRI ^10^ . Differences in judgment were resolved by consensus or by a third reviewer. Dissertations and theses were selected manually by reading the abstracts. Eligible studies were read in full by two reviewers, in a complementary manner, and validated by a third reviewer. The reference lists of included studies were checked to include other studies that might not have been retrieved in database searches.

### Data Extraction

A spreadsheet for extraction was prepared in Excel (Microsoft), containing the following information: (1) Author and year of publication, (2) Purpose, (3) Study design, (4) Population analyzed, (5) Number of participants, ( 6) Age of participants, (7) Sex/gender, (8) Race/color, (9) Family characteristics, (10) Country where the study was carried out, (11) Place where the study was carried out, (12) Focus of the approach and central theme, (13) Outcomes or thematic categories, (14) Results, (15) Limitations, (16) Gaps, (17) Conclusion, (18) Financing, (19) Conflict of interest, and (20) Institution of affiliation of the author. The first extractions were carried out independently by three reviewers, until homogeneity of the process was achieved. Subsequently, the data were extracted by two reviewers, in a complementary manner, and validated by a third reviewer.

### Data analysis

The extracted data was explored to present the state of the art regarding homoparental families in the cisgender population, seeking to report their needs and experiences related to the area of collective health. The results of the studies, mostly qualitative, were analyzed based on the content analysis technique adapted by Gomes ^11^ from the thematic modality described by Bardin ^12^ . The results are presented descriptively and through tables.

The methodological quality of the included studies was not assessed because it was not part of the inclusion criteria and is considered optional in scoping reviews ^7^ .

## RESULTS

The searches retrieved 1,350 records and, after excluding duplicates, 725 records were screened by titles and abstracts. Forty eligible reports were read in full, 24 of which were included. Of ten non-duplicated dissertations and theses, two were included. Additionally, 32 reports were selected from the reference lists of the included studies. Therefore, a total of 58 studies were included and analyzed in this scoping review ( Figure ). The sixteen studies and eight theses excluded are presented in OSF ^13^ .

**Figure:**
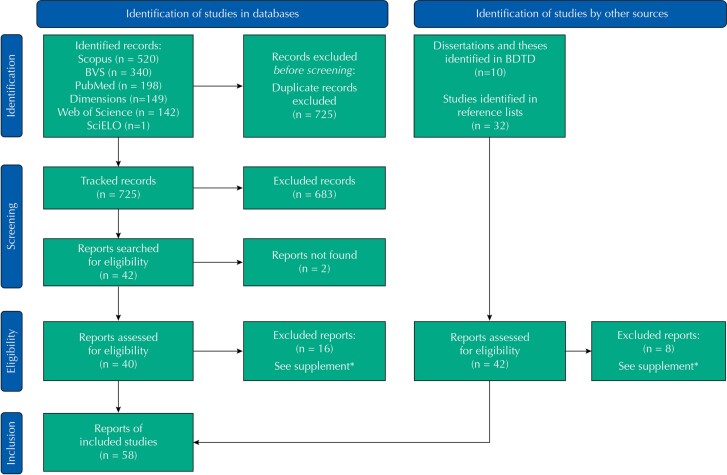
Study selection flowchart.

Source: based on Prisma ^14^ .

BDTD: Bank of Theses and Dissertations; VHL: Virtual Health Library; OSF: Open Science Framework.

* Excluded studies ^13^ .

Of 58 reports ^15^ , 40 were classified as primary studies (including two Brazilian master’s theses) ^54 , 61^ , 2 as essays, and 16 as reviews, whose characteristics are briefly described below.

### General Characteristics of Primary Studies

Chart 1 shows the main characteristics of primary studies. Regarding design, the studies are qualitative (n = 33), cross-sectional (n = 4), mixed (n = 2), and quantitative (n = 1). Most authors reported that they received financing (n = 19), while others did not receive it (n = 5) or did not report it (n = 16). Half of them reported having no conflict of interests and the other half did not report it.

**Chart 1:** General characteristics of primary studies.

Author	Study design	Study population	Age ( years)	Race/color/ethnicity	Country where it was held	Financing	Conflict of interest
Albuquerque et al. ^15^	Qualitative	Health professionals (Family Health Strategy nurses)	24–39, mean 30.3	Not shown	Brazil (Juazeiro do Norte, Ceará)	Not shown	Not shown
Andersen et al. ^16^	Qualitative	Lesbian, gay, or bisexual	33–49	Not shown	Sweden	Received no specific grant from any funding agency in the public, commercial or non-profit sectors	Declare that there is no conflict
Brennan and Sell ^17^	Qualitative	Lesbians and queers	27–44, mean 34	19 (95%) identified themselves as white	USA	Declare that there are no relevant financial relationships	Declare that there is no conflict
Carvalho et al. ^18^	Qualitative	Lesbians	27–43, mean 34	11 self-identified as white, 4 as mixed/brown, and 1 as black	Brazil (São Paulo and cities in the metropolitan region)	*Conselho Nacional de Desenvolvimento Científico e Tecnológico* (CNPq) and *Coordenação de Aperfeiçoamento de Pessoal de Nível Superior* (Capes)	Declare that there is no conflict
Chapman et al. ^19^	Qualitative	Lesbians, gay men, and transgenders	Not shown	Not shown	Australia	Nurses Memorial Trust of Western Australia and Channel 7 Telethon	Declare that there is no conflict
Chapman et al. ^20^	Qualitative	Lesbians	35–52	Not shown	Australia	Nurses Memorial Trust of Western Australia and Channel 7 Telethon	Declare that there is no conflict
Chapman et al. ^21^	Cross-sectional	Healthcare professionals (nurses and doctors)	27–48, mean 31.1 (doctors)	Caucasians: nurses (88.2%); doctors (61.1%)	Australia	Nurses Memorial Trust of Western Australia and Channel 7 Telethon	Declare that there is no conflict
			23–64, mean 40 (nurses)	Yellow : nurses (7.4%); doctors (33.3%)			
				Others: nurses (4.4%); doctors (5.6%)			
Dahl and Malterud ^24^	Qualitative	Lesbians	30–52	Not shown	Norway	The Norwegian Women’s Public Health Association	Declare that there is no conflict
Dibley ^25^	Qualitative	Lesbians	Not shown	Not shown	UK	Not shown	Not shown
Engström et al. ^27^	Qualitative	Lesbians	25–42, mean 34	Not shown	Sweden	Not shown	Declare that there is no conflict
Erlandsson et al. ^28^	Qualitative	Lesbians	26–48	Not shown	Sweden	Not shown	Not shown
Fantus ^29^	Qualitative	Gays and gestational surrogates (surrogacy)	Mean 39 (gay parents)	Gay parents: white: n = 13 (87%); yellow: n = 2 (13%)	Canada	Not shown	They declared that there was no conflict
			Mean 34 (gestational surrogates)	Gestational surrogates: white: n = 5 (83%); Aboriginal: n = 1 (17%)			
Goldberg et al. ^30^	Qualitative	Health professionals (perinatal nurses) and lesbians	30–40 (lesbian)	Not shown	Canada	Nova Scotia Health Research Foundation (NSHRF), in partnership with the Canadian Nurses Foundation Nursing Care Partnership Program	Not shown
			20–50 (nurses)				
Hayman et al. ^33^	Qualitative	Lesbians	28–58, mean 39.8	Not shown	Australia	Not shown	Not shown
Hayman et al. ^34^	Qualitative	Lesbians	28–58, mean 39.8	Not shown	Australia	Not shown	Not shown
Hayman and Wilkes ^35^	Qualitative	Lesbians	28–58, mean 39.8	Participants identified their cultural background as Australian (n = 21); Anglo-Australian (n = 2); Scottish-Australian (n = 2); Lebanese-Australian (n = 1); Maori-African-American-Australian (n = 1); Italian (n = 1); Dutch (n = 1); and Filipino (n = 1)	Australia	Not shown	Not shown
Juntareal and Spatz ^37^	Mixed	Lesbians	26–50, mean 34.5	White/Caucasian: n = 68 (100%).	USA	University of Pennsylvania, School of Nursing Student Grant	Declare that there is no conflict
				Black/African American: n = 2 (3%)			
				Hispanic/Latino: n = 1 (1%).			
				*Some interviewees selected more than one option			
Kerppola et al. ^39^	Qualitative	Lesbian, gay, bisexual, trans, or queer	Not shown	All participants were white and Finnish-speaking; some had an immigrant background	Finland	The authors did not receive financial support for the research	Declare no conflict
			Inclusion criteria: parents at least 18 years of age				
Klittmark et al. ^41^	Qualitative cross-sectional	Lesbian, gay, bisexual, trans, non-binary, and queer.	30–45	Whites	Sweden	Stiftelsen Einar Belven	Not shown
Larsson and Dykes ^42^	Qualitative	Lesbians	Not shown	Not shown	Sweden	Not shown	Not shown
Lee et al. ^44^	Qualitative	Lesbians	Not shown	Not shown	Scotland	Has not received a grant from any funding agency in the public, commercial or non-profit sectors	Declare that there is no conflict
Malmquist and Nelson ^46^	Qualitative	Lesbians	Mean 36	Not shown	Sweden	Not shown	Not shown
McNair et al. ^48^	Qualitative	Lesbians	29–62 (mothers)	Families of Anglo-Australian origins (n = 11); with Australian Aboriginal origins (n = 2); that contained members from southern Europe (n = 2); with northern European origins (n = 3); with Eastern European origins (n = 3); of Asian origin (n = 2); of Latin origin (n = 1)	Australia	Australian Research Council and Victorian Association of Family Therapists	Not shown
			4–34 (children)				
Mikhailovich et al. ^49^	Cross-sectional	Lesbians and gay men	Mean 38	Not shown	Australia	University of Canberra	Declare that there is no conflict
Nicol et al. ^50^	Cross-sectional	Health professionals in a pediatric tertiary hospital (nurses; doctors; other professions and employees)	Similar mean ages for nurses (37.1), physicians (35.9), and other healthcare professionals and other staff (37.4)	Caucasian: n = 178 (88.1%)	Australia	Nurses Memorial Trust of Western Australia	Declare that there is no conflict
				Others: n = 24 (11.9%)			
Nimbi et al. ^51^	Quantitative	Health professionals	Mean 34.54 (Sexology Educational Programs Group);	Not shown	Italy	Not shown	Declare that there is no conflict
			Mean 30 (Group without Educational Programs in Sexology)				
O’Neill et al. ^53^	Qualitative	Lesbians	Mid 30s–mid 40s	All women identified as being of European descent	New Zealand	Te Pou, New Zealand National Mental Health Research Center	Declare that there is no conflict
Obem ^54^	Qualitative (master’s thesis)	Lesbians and gay men	23–54	Not shown	Brazil (Rio Grande do Sul)	Capes	Not shown
Perrin and Kulkin ^55^	Qualitative	Lesbians and gay men	Not shown	White: n = 435 (94%)	USA	Joseph P. Healey Endowment Grant Award, 1993 to 1995	Not shown
				Hispanic: n = 11 (2%)			
				Black: n = 6 (1%)			
				Other/unknown: n = 15 (3%)			
Renaud ^56^	Qualitative and critical ethnography	Lesbians	20–40	Of the interviewees, Caucasian (n = 18), Hispanic (n = 2); and “woman of color,” as identified in the research (n = 1)	USA	Not shown	Not shown
				Support group participants: included “women of color”			
				Focus group: all were Caucasian			
Rondahl et al. ^57^	Qualitative	Lesbians	30–46	Not shown	Sweden	Uppsala University Hospital and Linkoping University, ISV/HAV	Declare that there is no conflict
Ross et al. ^58^	Qualitative	Lesbians or bisexuals	Not shown	Not shown	Canada	Lesbian and Gay Community Appeal Foundation of Toronto. Lori E. Ross is supported by a Career Scientist Award from the Ontario Ministry of Health and Long-Term Care and the Ontario Women’s Health Council. Leah Steele is supported as an academic researcher by the Department of Family Medicine, St. Michael’s Hospital, University of Toronto and the Health Systems Research and Consultancy Unit at the Center for Addiction and Mental Health, Toronto, Ontario, Canada; and, as a career scientist, by the Ontario Ministry of Health and Long-Term Care	Not shown
Rozental and Malmquist ^59^	Qualitative	Lesbians	26–45	Not shown	Sweden	Swedish Council for Working Life and Social Research	Not shown
Silva ^61^	Qualitative (master’s thesis)	Healthcare professionals and Lesbians	26–46 (lesbians)	Not shown	Brazil (states of Rio de Janeiro and São Paulo)	Not shown	Not shown
			29–60 (health professionals)				
Spidsberg ^64^	Qualitative hermeneutic phenomenological	Lesbians	Not shown	Not shown	Norway	Not shown	Not shown
Spidsberg and Sørlie ^65^	Qualitative	Health professionals (midwives)	30–59, mean 50	Not shown	Norway	The Norwegian Women’s Public Health Association	Declare that there is no conflict
Stewart ^66^	Qualitative	Lesbians	Not shown	White (100%)	UK	Not shown	Not shown
Doussa et al. ^67^	Qualitative	Lesbians and gay men; health and wellness service providers	Not shown	Not shown	Australia	Australian Research Council Linkage Grant, with financial support from the Victorian Health Promotion Foundation (VicHealth), Relationships Australia (National and Victoria), ACON (formerly the AIDS Council of NSW), and in-kind support from Gay and Lesbian Health Victoria and the Queensland Association for Healthy Communities	Declared that there was no conflict
Wilton and Kaufmann ^71^	Mixed	Lesbians	All except one were over 30 years old	White: n = 45 (100%)	UK	Not shown	Not shown
Wojnar and Katzenmeyer ^72^	Qualitative phenomenological descriptive	Lesbians	28–48, mean 37.2	White (n = 20), African American (n = 2), mixed ethnicity (n = 2)	Various communities in the Pacific Northwest	No relevant financing relationship	Declare that there is no conflict

These studies were carried out in Australia (n = 10), Sweden (n = 8), Brazil (n = 4), United States of America (n = 4), Canada (n = 3), Norway (n = 3), United Kingdom (n = 3), Scotland (n = 1), Finland (n = 1), Italy (n = 1), New Zealand (n = 1), and several communities in the Pacific Northwest (n = 1).

Most studies involved lesbian women (n = 32), gay men (n = 9), and healthcare professionals (n = 7). When provided, participants’ age ranged from 20 to 59 years old, with a predominance of white people.

### General Characteristics of Reviews and Essays

The main characteristics of the 2 essays and 16 reviews (2 systematic reviews, 1 meta-ethnography, 1 clinical guidelines review, 1 integrative review, 1 overview of reviews, and 10 narrative reviews) are shown in Chart 2 . The authors reported no conflict of interests (n = 9) or did not provide this information (n = 9). Some studies received financing (n = 5), others did not receive it (n = 3), but most did not provide this information (n = 10). The populations analyzed were lesbian women (n = 16), gay men (n = 7), and health professionals (n = 4).

**Chart 2:** General characteristics of reviews and essays.

Author	Type of review	Design of primary studies	Study population	Financing	Conflict of interest
Chapman et al. ^22^	Descriptive essay	Not shown	Health professionals	Nurses Memorial Trust of Western Australia; Channel 7 Telethon	Not shown
Dahl et al. ^23^	Metaethnography	13 empirical qualitative studies	Lesbian women	The Norwegian Women’s Public Health Association	Declare that there is no conflict
Eliason ^26^	Narrative review	Not shown	Lesbian and gay families; family nurses	Not shown	Not shown
Gregg ^31^	Review	10 qualitative studies	Lesbian women and healthcare professionals	There were no relevant financial relationships	Declare that there is no conflict
Hammond ^32^	Literature review	13 studies (does not provide design)	Lesbian mothers	Not shown	Not shown
Imaz ^36^	Essay with an anthropological approach	Assisted Human Reproduction Act	Gay and lesbian same-sex couples and families	Not shown	Reports no conflicts of financial or commercial interest
Kelsall-Knight ^38^	Literature review	Qualitative studies (n = 7); mixed methods (n = 1); quantitative studies, with a qualitative aspect (n = 2)	LGBT parents	Not shown	Declare that there is no conflict
Klein et al. ^40^	Review of clinical guidelines	17 clinical guidelines	LGBT	US Office of Population Affairs and Atlas Research	One of the researchers reports being on the advisory boards of Gilead Sciences, Inc. and Merck
Lee ^43^	Literature review	Not shown	lesbian mothers	Not shown	Not shown
Lucio and Araújo ^45^	Integrative review	5 descriptive studies with a qualitative approach	Lesbian women	Not shown	Not shown
McManus et al. ^47^	Literature review	15 articles (do not inform design)	Lesbian couples	Not shown	Not shown
Norton et al. ^52^	Documentary narrative review	Documents	Gay men who want to be fathers	Not shown	Declare that there is no conflict
Shields et al. ^60^	Systematic review	4 studies (2 quantitative studies with open questions for qualitative analysis and 2 qualitative studies that used semi-structured interviews)	LGBT parents	Nurses Memorial Trust and Channel 7 Telethon	Not shown
Silva et al. ^62^	Narrative review	Not shown	Same-sex couples	Not shown	Declare that there is no conflict
Singer ^63^	Narrative review	Not shown	Pregnant lesbians	Not shown	Not shown
Weber ^68^	Narrative review	Not shown	Lesbian and gay parents	Not shown	Not shown
Wells and Lang ^69^	Systematic literature review and metasynthesis	Qualitative interviews (n = 8); qualitative interviews with focus groups (n = 1); cross-sectional (n = 1)	Same-sex mothers; same-sex co-mothers; and midwives	Not shown	Declare that there is no conflict
Werner and Westerståhl ^70^	Review	Reviews (n = 5); interviews (n = 17); others (n = 2)	Lesbian couples	FoU (Research and Development) Södra Älvsborg	Declare that there is no conflict

### Mapping the Collection by Themes

When analyzing the collection of selected sources, we observed themes that were implicit or explicit in the contents of these sources ( Chart 3 ). Such themes are not necessarily exclusive. Some of them overlap and others are distinguished by their specificities.

**Chart 3:** Themes and respective subthemes covered in primary studies, theses, essays, and reviews.

Theme	Subthemes
Heteronormative context of health services ^15–18,21,24,26,29,30,32,34,39–43,48,51,54,56,57,59,65–68,72^	Compromised standard of healthcare of homosexual couples due to the hegemony of heterosexuality
	Inadequate forms and information systems for same-sex couples
	Discrimination against non-biological mothers or fathers
	Controversies about the fact that lesbians are mothers
	Not reducing parenting to blood ties
Disclosure of sexual orientation ^19,21–25,31,34,38,41,43,47–50,51,52,60,64,65,69,71^	Couples’ positions on non-disclosure because they do not consider it important
	Negative experiences of couples due to disclosure
	Defense of disclosure by couples because it can bring specific attention and demarcate a status to be recognized
	Medical professionals are less likely than nursing professionals to consider that there should be disclosure
Fertilization ^18^ ^,^ ^20^ ^,^ ^25^ ^,^ ^29^ ^,^ ^34^ ^,^ ^35^ ^,^ ^36^ ^,^ ^45^ ^,^ ^47^ ^,^ ^52^ ^,^ ^56^ ^,^ ^58^ ^,^ ^59^ ^,^ ^62^ ^,^ ^70^	Legal aspects
	Difficulty of access for homosexual couples
	Methods
	Male peers are more disadvantaged than female peers
	The legality of surrogacy varies in different countries
	Protocols on assisted reproduction do not cater for same-sex couples
	Regulation of in vitro fertilization makes access difficult for lesbians
	Priority for donor insemination techniques
	Prohibition of gay men donating sperm
	Future legal problems with the donor
	Measures for fertility services for homosexual couples
Perceptions of homosexuals and professionals about care provided and health services in general ^16–19,22,24,25,27,28,30,31–34,37,38,41,44,46,49,50,53–55,59,61,63,64,66–69,71,72^	Satisfactory care
	Unsatisfactory care
	Negative interactions
	Absence of emotional support
	Non-acceptance of non-biological mothers
	Homophobic comments
	Constraints and discrimination
	Lack of benefits for families headed by one or two homosexual adults
	Exclusive approach to the biological father
	Service denied
	Excessive curiosity of professionals
	Symbolic violence
	Service habits disturbed by the presence of homosexual couples
	Inappropriate language directed at homosexuals
	The need for an environment to protect oneself from homophobia
	Misunderstanding by professionals
	Professionals not prepared to care for homosexual couples
Information and educational actions ^27^ ^,^ ^32^ ^,^ ^39^ ^,^ ^41^ ^,^ ^51^ ^,^ ^71^	Positive score for sex education programs
	Insufficient information on induced lactation for non-biological mothers
	Information exclusively heterosexual in nature
	Registration denied in educational group

The data extracted from the studies were grouped into five themes, presented together with their respective subthemes in Chart 3 . Perceptions of homosexuals and professionals regarding care provided and health services in general was the theme addressed by the largest number of studies (n = 31), followed by the heteronormative context of health services (n = 26); disclosure of sexual orientation (n = 20); fertilization (n = 14); and information and educational actions (n = 5).

## DISCUSSION

The scientific production in the health sector in general regarding homo-parenthood appears to be an issue whose approach requires the understanding of socio-structural aspects that go beyond this field of knowledge. At least two of these aspects can be highlighted. The first of these concerns heteronormativity, which, in a hegemonic way, means that—consciously or unconsciously—the first reference we have to family or parenting involves the union of a cis man with a cis woman. The existence of a homosexual couple means that this heterosexual norm is either reaffirmed to disqualify such a couple or deconstructed to accept homoaffective unions and parents. In this sense, it appears that much of the reviewed literature, before dealing with specific objects related to homoparenting, mentions the heteronormative context both as an explanatory model for the non-existence of specific health actions for lesbian or gay couples and as a dimension to be questioned or relativized as a unique reference to demand differentiated attention for these couples.

Another aspect that emerges in the reviewed scientific production, which covers issues that go beyond the health area in dealing with same-sex parenthood, refers to the legislation, or lack thereof, that ensures or prohibits not only the union of same-sex persons but also the desire of these persons to have children. Such aspects, directly or indirectly, are associated with the heteronormative context. We observed that, regarding legal aspects, there is great variability between countries and even within the states that make up a country. The absence of legal provisions, their incompleteness and/or dubiousness directly reflect on the way couples are assisted or are unable to access care.

Disclosing sexual orientation, both from the perspective of homosexual couples and from health professionals, emerges in the literature as something controversial. On the one hand, disclosure can contribute to specific health actions aimed at such couples. On the other hand, according to some studies, in the perception of lesbians and gays, disclosure can result in discrimination, invasive questioning, prejudice and even symbolic violence. The fear of disclosing homosexuality, in a certain way, can be linked to the heteronormative context and legal issues.

Scientific production on fertilization involves issues related to legislation, rights, access difficulties, absence or insufficient information, exclusion of non-biological homosexual mothers or fathers, prenatal care, childbirth, postpartum, and methods. The literature that deals with this topic focuses mainly on lesbians. In the balance made in the results of the studies, difficulties in accessing fertilization technology predominate.

Perceptions regarding attention to homoparenting, on the part of both homosexual couples and health professionals, are generally linked to the existence of dissatisfaction with the care received and negative attitudes on the part of those who should provide adequate care.

Regarding information and educational actions, the literature reports some positive experiences. However, these experiences compete with the perception that information is insufficient. Still in educational terms, there is an issue that crosses all the themes identified, explicitly or implicitly in the results: the lack of health professionals’ preparation to deal not only with homo-parenthood, but also with homosexuality.

The revised collection constitutes a mosaic of themes that, directly or indirectly, are related to same-sex parenting. Each one of them, either by what is explicit or by inference of what is implicit, can provide principles for the field of collective health. In this sense, the results of this review are important, since they provide elements for, among other aspects, the organization of health services, the implementation of specific actions within the scope of promoting family health, and the adequate training of professionals to address gay and lesbian families.

It is also observed that the mapping obtained regarding the scope of the study is a starting point to expand the discussion about the central theme. This expansion may be more successful to the extent that, anchored in socio-anthropological references, it can problematize issues focused on different family arrangements and other conceptions of kinship that are not limited to consanguinity.

Finally, it is highlighted that, despite the vast collection identified, a limitation that can be pointed out for this review is language filtering, choosing only sources in Portuguese, Spanish, and English. Particularly noteworthy is the lack of studies in the French language, which gave rise to the term homo-parenthood. In addition to this, the bases chosen for the research may also have influenced the lack of studies in French.

## CONCLUSIONS

Among the main conclusions it is worth highlighting that, although the national literature on homoparenting in the health sector is still timid, the international discussion seems to be relatively expanding. In terms of evidence, we can highlight that the scope of this review is not sufficiently problematized in health professionals’ training and performance; and quantitative studies are smaller in number compared to those of a qualitative nature. This, although it brings us the specificities of the central theme, does not allow us to understand the extent of the problem highlighted in most studies.

Mapping the literature on the subject also revealed some gaps in the scientific production reviewed. In the context of collective health, it is worth highlighting the lack of studies focused on policies and programs and the absence of discussions on the health of children and adolescents from homo-parental families.
