# Supplementary material for: Homoparenting as a public health issue: a scoping review
Source: Rev Saude Publica. 2023 Oct 24;57:80. doi: 10.11606/s1518-8787.2023057005447 (PMC10609659; doi:10.11606/s1518-8787.2023057005447)
Supplement: Translation

# Translation: A homoparentalidade como questão da saúde coletiva: uma revisão de escopo

**Authors:** Romeu Gomes, Tereza Setsuko Toma, Jessica De Lucca Da Silva, Fernando Meirinho Domene, Adriano da Silva

**Affiliations:** I Brasil Fundação Oswaldo Cruz. Instituto Nacional de Saúde da Mulher, da Criança e do Adolescente Fernandes Figueira. Departamento de Ensino. Rio de Janeiro, RJ, Brasil; II Brasil Hospital Sírio-Libanês. Diretoria de Compromisso Social. São Paulo, SP, Brasil .; III Brasil Secretaria de Estado da Saúde de São Paulo. Instituto de Saúde (NEv-IS). Núcleo de Evidências. São Paulo, SP, Brasil; IV Brasil Fundação Oswaldo Cruz. Escola Nacional de Saúde Pública Sergio Arouca. Departamento de Estudos sobre Violência e Saúde Jorge Careli. Rio de Janeiro, RJ, Brasil

**Keywords:** Homossexualidade, Família, Minorias Sexuais e de Gênero, Saúde, Revisão

## Abstract

**OBJETIVO:**

Mapear a produção científica global sobre homoparentalidade no campo da saúde coletiva ou saúde pública.

**MÉTODOS:**

Em termos de procedimentos metodológicos, foi realizada uma revisão de escopo, tendo como norte a seguinte pergunta: quais são os aspectos abordados na produção científica global a respeito de famílias homoparentais no campo da saúde coletiva ou pública? As buscas foram realizadas em sete fontes de literatura científica, sendo incluídos 58 estudos, envolvendo artigos científicos e dissertações. O tratamento analítico dado aos estudos, em sua maioria qualitativos, seguiu a técnica de análise de conteúdo na modalidade temática.

**RESULTADOS:**

Os resultados indicam que percepções de homossexuais e de profissionais sobre cuidados prestados e serviços de saúde em geral foi a temática abordada por maior número de estudos (n = 31), seguida de contexto heteronormativo dos serviços de saúde (n = 26); revelação da orientação sexual (n = 20); fertilização (n = 14); informações e ações educativas (n = 5).

**CONCLUSÃO:**

Embora a questão da homoparentalidade venha sendo discutida em alguns setores da saúde, há ciência de que é preciso contar com uma base consolidada por meio de inúmeros estudos ao se problematizar essa temática. Conclui-se que, dentre outros aspectos, que o escopo desta revisão não é problematizado de forma suficiente no âmbito da formação e atuação de profissionais de saúde.

## INTRODUÇÃO

A família tem sido um dos focos centrais em diversas instâncias da saúde coletiva. A exemplo disso, destaca-se a Estratégia da Saúde da Família, um dos modelos de organização dos serviços no Sistema Único de Saúde (SUS). Nessa e em outras instâncias, a referência de família comumente utilizada é o modelo tradicional, que se origina da união entre um homem e uma mulher cis. Essa união institui, nos contextos, nos textos e nas relações da área da saúde em geral, a hegemonia da parentalidade heterossexual, desconsiderando a homoparentalidade ou parentalidade homoafetiva, que é o tema deste artigo.

Para que se possa discutir a homoparentalidade, faz-se necessário – com base em estudos antropológicos – levar em conta que os tipos de relacionamentos tidos como família podem ser vistos de formas diferenciadas dentro de suas próprias sociedades, não se limitando a relacionamentos definidos genealogicamente^1^. Levando em conta os diferentes tipos de relacionamentos, as famílias de gays e lésbicas podem abranger amantes, coparentalidade, filhos adotivos, filhos de relacionamento anterior e filhos concebidos por meio de inseminação alternativa^1^.

Embora a questão da homoparentalidade venha sendo discutida em alguns setores da saúde, há ciência de que é preciso contar com uma base consolidada por meio de inúmeros estudos ao se problematizar essa temática. Uma das dimensões a serem contempladas é formar um quadro analítico, à luz da literatura especializada, que possa servir de referência para a inserção da discussão acerca do objeto de estudo tanto nas lógicas quanto nos cenários das práticas da saúde coletiva.

Zambrano^2^observa que:

Homoparentalidade é um neologismo criado em 1997 pela Associação de Pais e Futuros Pais Gays e Lésbicas (APGL), em Paris, nomeando a situação na qual pelo menos um adulto que se autodesigna homossexual é (ou pretende ser) pai ou mãe de, no mínimo, uma criança (p. 127)^2^.

Ribeiro et al.^3^(p. 3592), com base em Zambrano^2^, observam que a homoparentalidade se constitui a partir de, pelo menos, quatro situações:

[...] por filhos havidos em uma ligação heterossexual anterior, pela adoção legal ou informal, através de usos das novas tecnologias reprodutivas que possibilitam o nascimento de filhos biológicos, e pela coparentalidade, na qual os cuidados com a criança são exercidos de forma conjunta e igualitária pelos parceiros^3^(p. 3592).

Com o intuito de situar a homoparentalidade no contexto das mudanças que vêm ocorrendo na instituição família, observa-se que a família patriarcal vem sendo posta em questão desde o final do último milênio. A dissociação entre heterossexualidade, patriarcalismo e reprodução da espécie reforçou a luta do movimento gay e lésbico nos últimos anos, para ter o reconhecimento legal de casar-se, formar família e ter filhos^4^.

Em consonância com esse pleito, a exclusividade de haver um homem e uma mulher cis para constituir o que se denomina família é questionada, de modo que, se o vínculo da afetividade for considerado como central da instituição familiar, a união entre pessoas do mesmo sexo pode ser considerada como família^5^.

Apesar de a discussão do assunto não ser nova, infere-se que – na área da saúde em geral do Brasil – as publicações sobre homoparentalidade são escassas. Um levantamento conciso, realizado no dia 7 de julho de 2021, com as expressões “homoparentalidade *AND* saúde”, localizou apenas dois artigos na *Scientific Electronic Library Online* (SciELO) e quatro no Portal Regional da Biblioteca Virtual em Saúde (BVS).

Evidentemente que esses rápidos levantamentos não representam o estado da arte da temática no âmbito da produção científica brasileira, sendo necessárias buscas mais aprofundadas, de forma sistemática, com uma grande abrangência de bases de dados.

Nesse sentido, propõe-se realizar uma revisão de escopo, com o objetivo de mapear a produção científica global sobre homoparentalidade no campo da saúde coletiva ou saúde pública.

No Brasil e em alguns países latino-americanos, há uma diferença entre saúde coletiva e saúde pública. A primeira expressão, segundo Paim^6^, é um campo integrado por saber, prática e ideologia, diferenciando-se tanto da saúde pública quanto do modelo médico hegemônico e articulando a ciência e práticas para a formulação e condução de políticas consequentes. Assim, o coletivo não é apenas uma população ou segmento populacional abstrato, e as ações voltadas para o coletivo não são de exclusividade do Estado. No panorama internacional, em geral, não aparece o termo saúde coletiva, e sim saúde pública, que abrange medidas concebidas e adotadas principalmente pelo Estado para assegurar o bem-estar físico, mental e social da população. Nesse sentido, analisa-se o escopo desta revisão no âmbito da saúde coletiva ou da saúde pública para que não se reduza a produção ao âmbito latino-americano.

## MÉTODOS

Realizamos uma revisão de escopo com base no referencial metodológico do *Joanna Briggs Institute* ^7^. Para o relato desta revisão foram utilizadas as recomendações da ferramenta *PRISMA Extension for Scoping Reviews* ^8^. Um protocolo de pesquisa foi registrado na *Open Science Framework* (OSF)^9^.

### Pergunta de Investigação

A pergunta “Quais são os aspectos abordados na produção científica global a respeito de famílias homoparentais no campo da saúde coletiva ou pública?” foi construída com auxílio do acrônimo PCC (População: famílias homoparentais cisgêneras; Conceito: produção científica global; Contexto: saúde coletiva ou pública). Optou-se por trabalhar com uma pergunta aberta e ampla para obter uma maior diversidade da produção científica acerca do assunto.

### Critérios de Inclusão e Exclusão

Os critérios de inclusão foram estudos primários e secundários, entre documentos, relatórios, dissertações ou teses, disponíveis em inglês, português ou espanhol, que abordaram questões relacionadas a políticas, programas de saúde e acesso a serviços para famílias homoparentais cisgêneras no contexto da saúde coletiva ou pública.

Foram excluídos estudos que se referiam a outros contextos que não a saúde coletiva, que analisaram configurações de famílias homoparentais não cisgêneras ou que estavam em idiomas diferentes dos citados acima.

### Fontes de Dados e Estratégias de Busca

A construção das estratégias e as buscas foram realizadas por um bibliotecário nas seguintes fontes de dados: PubMed/MEDLINE, Literatura Latino-Americana e do Caribe em Ciências da Saúde na Biblioteca Virtual em Saúde (BVS/LILACS), SciELO, Scopus, *Web of Science, Dimensions* (julho de 2022) e Biblioteca Digital Brasileira de Teses e Dissertações (BDTD) (setembro de 2022). Com base na combinação de palavras-chave estruturadas a partir do acrônimo PCC, foram utilizados os termos MeSH ( *Medical Subject Headings* ) no PubMed e DeCS (Descritores em Ciências da Saúde) na BVS, adaptando-os para as demais bases de dados. As estratégias de busca com os descritores utilizados em cada base estão disponíveis no protocolo desta revisão registrado em OSF^9^.

### Seleção dos Estudos

Os estudos recuperados das fontes de informação passaram por um processo de seleção com base nos critérios de inclusão e exclusão pré-definidos. Após a exclusão de duplicatas, dois revisores realizaram, de modo independente, a triagem com base na leitura de títulos e resumos, utilizando o gerenciador bibliográfico *Rayyan QCRI* ^10^. As divergências de julgamento foram resolvidas por consenso ou por um terceiro revisor. A seleção de dissertações e teses foi realizada manualmente por meio da leitura dos resumos. Os estudos elegíveis foram lidos na íntegra por dois revisores, de modo complementar, e validados por um terceiro revisor. As listas de referências dos estudos incluídos foram verificadas para inclusão de outros estudos eventualmente não recuperados nas buscas em bases de dados.

### Extração dos Dados

Uma planilha para extração foi elaborada no *software* Excel, contendo as seguintes informações: (1) Autor e ano de publicação, (2) Objetivo, (3) Delineamento do estudo, (4) População analisada, (5) Número de participantes, (6) Idade dos participantes, (7) Sexo/gênero, (8) Raça/cor, (9) Características da família, (10) País de realização do estudo, (11) Local de realização do estudo, (12) Foco da abordagem e tema central, (13) Desfechos ou categorias temáticas, (14) Resultados, (15) Limitações, (16) Lacunas, (17) Conclusão, (18) Financiamento, (19) Conflito de interesse e (20) Instituição de filiação do(a) autor(a). As primeiras extrações foram realizadas, de modo independente, por três revisores, até se chegar a uma homogeneidade do processo. Posteriormente, os dados foram extraídos por dois revisores, de modo complementar, e validados por um terceiro revisor.

### Análise dos Dados

Os dados extraídos foram explorados para apresentar o estado da arte acerca de famílias homoparentais na população cisgênera, buscando relatar suas necessidades e experiências relacionadas à área de saúde coletiva. Os resultados dos estudos, em sua maioria qualitativos, foram analisados à luz da técnica de análise de conteúdo adaptada por Gomes^11^da modalidade temática descrita por Bardin^12^. Os resultados são apresentados de forma descritiva e por meio de quadros.

Não se realizou avaliação da qualidade metodológica dos estudos incluídos, uma vez que ela não fez parte dos critérios de inclusão, sendo considerada opcional em revisões de escopo^7^.

## RESULTADOS

As buscas recuperaram 1.350 registros e, após exclusão de duplicatas, 725 registros foram triados por títulos e resumos. Quarenta relatos elegíveis foram lidos na íntegra, sendo 24 incluídos. De dez dissertações e teses não duplicadas, duas foram incluídas. Adicionalmente, 32 relatos foram selecionados das listas de referências dos estudos incluídos. Desse modo, no total 58 estudos foram incluídos e analisados nesta revisão de escopo ( Figura ). Os dezesseis estudos e oito teses excluídos são apresentados na OSF^13^.

**Figura:** Fluxograma da seleção de estudos.

Fonte: baseado no Prisma ^14^ .

BDTD: Banco de Teses e Dissertações; BVS: Biblioteca Virtual em Saúde; OSF: *Open Science Framework* .

* Estudos excluídos ^13^ .

De 58 relatos^15^, 40 foram classificados como estudos primários (incluindo duas dissertações de mestrado brasileiras)^54 , 61^, 2 ensaios e 16 revisões, cujas características são descritas brevemente a seguir.

### Características Gerais dos Estudos Primários

As principais características dos estudos primários são apresentadas no Quadro 1 . Quanto ao delineamento, os estudos são qualitativos (n = 33), transversais (n = 4), mistos (n = 2) e quantitativo (n = 1). Os autores relataram que receberam financiamento (n = 19), não receberam (n = 5), ou não informaram (n = 16). Metade deles relatou não ter conflito de interesses e a outra metade não informou.

**Quadro 1:** Características gerais dos estudos primários.

Autor	Delineamento do estudo	População do estudo	Idade (anos)	Raça/cor/etnia	País onde foi realizado	Financiamento	Conflito de interesse
Albuquerque et al. ^15^	Qualitativo	Profissionais de saúde (enfermeiros da Estratégia Saúde da Família)	24–39, média de 30,3	Não informado	Brasil (Juazeiro do Norte, Ceará)	Não informado	Não informado
Andersen et al. ^16^	Qualitativo	Lésbicas, gays ou bissexuais	33–49	Não informado	Suécia	Não recebeu concessão específica de nenhuma agência de financiamento nos setores público, comercial ou sem fins lucrativos	Declaram não haver conflito
Brennan e Sell ^17^	Qualitativo	Lésbicas e queers	27–44, média de 34	19 (95%) se identificaram como brancas	Estados Unidos da América	Declaram não haver relações financeiras relevantes	Declaram não haver conflito
Carvalho et al. ^18^	Qualitativo	Lésbicas	27–43, média de 34	11 se autoidentificaram como brancas, 4 como pardas e 1 como preta	Brasil (São Paulo e cidades da região metropolitana)	Conselho Nacional de Desenvolvimento Científico e Tecnológico (CNPq) e Coordenação de Aperfeiçoamento de Pessoal de Nível Superior (Capes)	Declaram não haver conflito
Chapman et al. ^19^	Qualitativo	Lésbicas, gays e transgêneros	Não informado	Não informado	Austrália	Nurses Memorial Trust of Australia Ocidental e Canal 7 Telethon	Declaram não haver conflito
Chapman et al. ^20^	Qualitativo	Lésbicas	35–52	Não informado	Austrália	Nurses Memorial Trust of Western Australia e Channel 7 Telethon	Declaram não haver conflito
Chapman et al. ^21^	Transversal	Profissionais de saúde (enfermeiros e médicos)	27–48, média de 31,1 (médicos)	Caucasianos: enfermeiras (88,2%); médicos (61,1%)	Austrália	Nurses Memorial Trust of Western Australia e Channel 7 Telethon	Declaram não haver conflito
			23–64, média de 40 (enfermeiros)	Amarelos: enfermeiras (7,4%); médicos (33,3%)			
				Outras: enfermeiras (4,4%); médicos (5,6%)			
Dahl e Malterud ^24^	Qualitativo	Lésbicas	30–52	Não informado	Noruega	The Norwegian Women’s Public Health Association	Declaram não haver conflito
Dibley ^25^	Qualitativo	Lésbicas	Não informado	Não informado	Reino Unido	Não informado	Não informado
Engström et al. ^27^	Qualitativo	Lésbicas	25–42, média de 34	Não informado	Suécia	Não informado	Declaram não haver conflito
Erlandsson et al. ^28^	Qualitativo	Lésbicas	26–48	Não informado	Suécia	Não informado	Não informado
Fantus ^29^	Qualitativo	Gays e substitutos gestacionais (barrigas de aluguel)	Média de 39 (pais gays)	Pais gays: branco: n = 13 (87%); amarelo: n = 2 (13%)	Canadá	Não informado	Declararam não haver conflito
			Média de 34 (substitutos gestacionais)	Substitutos gestacionais: branco: n = 5 (83%); aborígene: n = 1 (17%)			
Goldberg et al. ^30^	Qualitativo	Profissionais de saúde (enfermeiras perinatais) e lésbicas	30–40 (lésbicas)	Não informado	Canadá	Nova Scotia Health Research Foundation (NSHRF), em parceria com o Canadian Nurses Foundation Nursing Care Partnership Program	Não informado
			20–50 (enfermeiras)				
Hayman et al. ^33^	Qualitativo	Lésbicas	28–58, média de 39,8	Não informado	Austrália	Não informado	Não informado
Hayman et al. ^34^	Qualitativo	Lésbicas	28–58, média de 39,8	Não informado	Austrália	Não informado	Não informado
Hayman e Wilkes ^35^	Qualitativo	Lésbicas	28–58, média de 39,8	As participantes identificaram sua formação cultural como australiana (n = 21); anglo-australiana (n = 2); escocês-australiana (n = 2); libanês-australiana (n = 1); maori-afro-americana- australiana (n = 1); italiana (n = 1); holandesa (n = 1); e filipina (n = 1)	Austrália	Não informado	Não informado
Juntereal e Spatz ^37^	Misto	Lésbicas	26–50, média de 34,5	Branco/caucasiano: n = 68 (100%).	Estados Unidos da América	University of Pennsylvania, School of Nursing Student Grant	Declaram não haver conflito
				Negro/afro-americano: n = 2 (3%)			
				Hispânico/latino: n = 1 (1%).			
				*Algumas entrevistadas selecionaram mais de uma opção			
Kerppola et al. ^39^	Qualitativo	Lésbicas, gays, bissexuais, trans ou queer	Não informado	Todos os participantes eram brancos e falantes de finlandês; alguns tinham origem imigrante	Finlândia	Os autores não receberam apoio financeiro para a pesquisa	Declaram não haver conflito
			Critério de inclusão: pais com pelo menos 18 anos de idade				
Klittmark et al. ^41^	Transversal qualitativo	Lésbicas, gays, bissexuais, trans, não-binários e queer.	30–45	Brancos	Suécia	Stiftelsen Einar Belven	Não informado
Larsson e Dykes ^42^	Qualitativo	Lésbicas	Não informado	Não informado	Suécia	Não informado	Não informado
Lee et al. ^44^	Qualitativo	Lésbicas	Não informado	Não informado	Escócia	Não recebeu concessão de nenhuma agência de financiamento nos setores público, comercial ou sem fins lucrativos	Declaram não haver conflito
Malmquist e Nelson ^46^	Qualitativo	Lésbicas	Média de 36	Não informado	Suécia	Não informado	Não informado
McNair et al. ^48^	Qualitativo	Lésbicas	29–62 (mães)	Famílias de origens anglo-australianas (n = 11); com origens aborígenes australianas (n = 2); que continham membros do sul da Europa (n = 2); com origens do norte da Europa (n = 3); com origens do Leste Europeu (n = 3); de origem asiática (n = 2); de origem latina (n = 1)	Austrália	Australian Research Council e Victorian Association of Family Therapists	Não informado
			4–34 (filhos)				
Mikhailovich et al. ^49^	Transversal	Lésbicas e gays	Média de 38	Não informado	Austrália	University of Canberra	Declaram não haver conflito
Nicol et al. ^50^	Transversal	Profissionais de saúde em hospital terciário pediátrico (enfermeiros; médicos; outras profissões e funcionários)	Idades médias semelhantes para enfermeiros (37,1), médicos (35,9) e outros profissionais de saúde e outros funcionários (37,4)	Caucasiano: n = 178 (88,1%)	Austrália	Nurses Memorial Trust of Western Australia	Declaram não haver conflito
				Outros: n = 24 (11,9%)			
Nimbi et al. ^51^	Quantitativo	Profissionais de saúde	Média de 34,54 (Grupo Programas Educacionais em Sexologia);	Não informado	Itália	Não informado	Declaram não haver conflito
			média de 30 (Grupo sem Programas Educacionais em Sexologia)				
O’Neill et al. ^53^	Qualitativo	Lésbicas	Meados de 30–meados de 40	Todas as mulheres se identificaram como descendentes de europeus	Nova Zelândia	Te Pou, Centro Nacional de Pesquisa em Saúde Mental da Nova Zelândia	Declaram não haver conflito
Obem ^54^	Qualitativo (dissertação de mestrado)	Lésbicas e gays	23–54	Não informado	Brasil (Rio Grande do Sul)	Capes	Não informado
Perrin e Kulkin ^55^	Qualitativo	Lésbicas e gays	Não informado	Branca: n = 435 (94%)	Estados Unidos da América	Prêmio Joseph P. Healey Endowment Grant, de 1993 a 1995	Não informado
				Hispânico: n = 11 (2%)			
				Preto: n = 6 (1%)			
				Outro/desconhecido: n = 15 (3%)			
Renaud ^56^	Qualitativo e etnografia crítica	Lésbicas	20–40	Das entrevistadas, caucasianas (n = 18), hispânicas (n = 2); e “mulher de cor”, conforme identificação constante da pesquisa (n = 1)	Estados Unidos da América	Não informado	Não informado
				Participantes do grupo de apoio: incluíam “mulheres de cor”			
				Grupo focal: todas eram caucasianas			
Röndahl et al. ^57^	Qualitativo	Lésbicas	30–46	Não informado	Suécia	Uppsala University Hospital e Linköping University, ISV/HAV	Declaram não haver conflito
Ross et al. ^58^	Qualitativo	Lésbicas ou bissexuais	Não informado	Não informado	Canadá	Lesbian and Gay Community Appeal Foundation of Toronto. Lori E. Ross é apoiada por um Prêmio Cientista de Carreira do Ministério da Saúde e Cuidados de Longo Prazo de Ontário e do Conselho de Saúde da Mulher de Ontário. Leah Steele é apoiada como pesquisadora acadêmica do Departamento de Medicina Familiar do Hospital St. Michael, da Universidade de Toronto e da Unidade de Consultoria e Pesquisa de Sistemas de Saúde do Centro de Dependência e Saúde Mental, Toronto, Ontário, Canadá; e, como cientista de carreira, pelo Ministério da Saúde e Cuidados de Longo Prazo de Ontário	Não informado
Rozental e Malmquist ^59^	Qualitativo	Lésbicas	26–45	Não informado	Suécia	Swedish Council for Working Life and Social Research	Não informado
Silva ^61^	Qualitativo (dissertação de mestrado)	Profissionais de saúde e lésbicas	26–46 (lésbicas)	Não informado	Brasil (estados do Rio de Janeiro e São Paulo)	Não informado	Não informado
			29–60 (profissionais da saúde)				
Spidsberg ^64^	Qualitativo hermenêutico fenomenológico	Lésbicas	Não informado	Não informado	Noruega	Não informado	Não informado
Spidsberg e Sørlie ^65^	Qualitativo	Profissionais de saúde (parteiras)	30–59, média de 50	Não informado	Noruega	The Norwegian Women’s Public Health Association	Declaram não haver conflito
Stewart ^66^	Qualitativo	Lésbicas	Não informado	Brancas (100%)	Reino Unido	Não informado	Não informado
Doussa et al. ^67^	Qualitativo	Lésbicas e gays; prestadores de serviços de saúde e bem-estar	Não informado	Não informado	Austrália	Australian Research Council Linkage Grant, com apoio financeiro da Victorian Health Promotion Foundation (VicHealth), Relationships Australia (National e Victoria) e ACON (anteriormente AIDS Council of NSW) e apoio em espécie de Gays e Lésbicas Health Victoria e a Associação de Queensland para Comunidades Saudáveis	Declararam não haver conflito
Wilton e Kaufmann ^71^	Misto	Lésbicas	Todas, exceto uma, tinham mais de 30 anos	Brancas: n = 45 (100%)	Reino Unido	Não informado	Não informado
Wojnar e Katzenmeyer ^72^	Qualitativo fenomenológico descritivo	Lésbicas	28–48, média de 37,2	Branca (n = 20), afro-americanas (n = 2), etnia mista (n = 2)	Várias comunidades no noroeste do Pacífico	Nenhuma relação relevante de financiamento	Declaram não haver conflito

Esses estudos foram realizados na Austrália (n = 10), Suécia (n = 8), Brasil (n = 4), Estados Unidos da América (n = 4), Canadá (n = 3), Noruega (n = 3), Reino Unido (n = 3), Escócia (n = 1), Finlândia (n = 1), Itália (n = 1), Nova Zelândia (n = 1) e várias comunidades no noroeste do Pacífico (n = 1).

A maioria dos estudos envolveu mulheres lésbicas (n = 32), homens gays (n = 9) e profissionais de saúde (n = 7). Quando informada, a idade dos participantes variou de 20 a 59 anos, com predominância da cor branca.

### Características Gerais das Revisões e Ensaios

As principais características dos 2 ensaios e 16 revisões (2 revisões sistemáticas, 1 meta-etnografia, 1 revisão de diretrizes clínicas, 1 revisão integrativa, 1 revisão de revisões e 10 revisões narrativas) são apresentadas no Quadro 2 . Os autores relataram não ter conflito de interesses (n = 9) ou não apresentaram essa informação (n = 9). Os estudos receberam financiamento (n = 5), não receberam (n = 3) ou não trouxeram essa informação (n = 10). As populações analisadas foram mulheres lésbicas (n = 16), homens gays (n = 7) e profissionais de saúde (n = 4).

**Quadro 2:** Características gerais das revisões e ensaios.

Autor	Tipo de revisão	Delineamento dos estudos primários	População do estudo	Financiamento	Conflito de interesse
Chapman et al. ^22^	Ensaio descritivo	Não informado	Profissionais de saúde	Nurses Memorial Trust of Western Australia; Channel 7 Telethon	Não informado
Dahl et al. ^23^	Metaetnografia	13 estudos qualitativos empíricos	Mulheres lésbicas	The Norwegian Women’s Public Health Association	Declaram não haver conflito
Eliason ^26^	Revisão narrativa	Não informado	Famílias de lésbicas e gays; enfermeiras(os) de família	Não informado	Não informado
Gregg ^31^	Revisão	10 estudos qualitativos	Mulheres lésbicas e profissionais da saúde	Não houve relações financeiras relevantes	Declara não haver conflito
Hammond ^32^	Revisão de literatura	13 estudos (não informa delineamento)	Mães lésbicas	Não informado	Não informado
Imaz ^36^	Ensaio com abordagem antropológica	Lei de Reprodução Humana Assistida	Casais e famílias homoafetivas de gays e lésbicas	Não informado	Relata não ter conflitos de interesse financeiro ou comercial
Kelsall-Knight ^38^	Revisão de literatura	Estudos qualitativos (n = 7); métodos mistos (n = 1); estudos quantitativos, com um aspecto qualitativo (n = 2)	Pais LGBT	Não informado	Declara não haver conflito
Klein et al. ^40^	Revisão de diretrizes clínicas	17 diretrizes clínicas	LGBT	U.S. Office of Population Affairs and Atlas Research	Um dos pesquisadores relata estar nos conselhos consultivos da Gilead Sciences, Inc. e da Merck
Lee ^43^	Revisão de literatura	Não informado	Mães lésbicas	Não informado	Não informado
Lucio e Araújo ^45^	Revisão integrativa	5 estudos descritivos com abordagem qualitativa	Mulheres lésbicas	Não informado	Não informado
McManus et al. ^47^	Revisão de literatura	15 artigos (não informam delineamento)	Casais de lésbicas	Não informado	Não informado
Norton et al. ^52^	Revisão narrativa documental	Documentos	Homens gays que querem ser pais	Não informado	Declaram não haver conflito
Shields et al. ^60^	Revisão sistemática	4 estudos (2 estudos quantitativos com perguntas abertas para análise qualitativa e 2 estudos qualitativos que utilizaram entrevistas semiestruturadas)	Pais/mães LGBT	Nurses Memorial Trust e Channel 7 Telethon	Não informado
Silva et al. ^62^	Revisão narrativa	Não informado	Casais homoafetivos	Não informado	Declaram não haver conflito
Singer ^63^	Revisão narrativa	Não informado	Lésbicas grávidas	Não informado	Não informado
Weber ^68^	Revisão narrativa	Não informado	Pais/mães gays e lésbicas	Não informado	Não informado
Wells e Lang ^69^	Revisão sistemática de literatura e metassíntese	Entrevistas qualitativas (n = 8); entrevistas qualitativas com grupos focais (n = 1); transversal (n = 1)	Mães do mesmo sexo; comães do mesmo sexo; e parteiras	Não informado	Declaram não haver conflito
Werner e Westerståhl ^70^	Revisão	Revisões (n = 5); entrevistas (n = 17); outros (n = 2)	Casais de lésbicas	FoU (Pesquisa e Desenvolvimento) Södra Älvsborg	Declaram não haver conflito

### Mapeamento do Acervo por Temáticas

Ao analisarmos o acervo das fontes selecionadas, observamos temáticas que estavam implícitas ou explícitas nos conteúdos dessas fontes ( Quadro 3 ). Tais temáticas não são necessariamente excludentes. Algumas delas se sobrepõem e outras se distinguem pelas suas especificidades.

**Quadro 3:** Temáticas e respectivos subtemas abordados nos estudos primários, teses, ensaios e revisões.

Temática	Subtemas
Contexto heteronormativo dos serviços de saúde^15–18, 21,24,26,29,30,32,34,39–43,48,51,54,56,57,59,65–68,72^	Comprometimentos nos cuidados de saúde de casais homossexuais por conta da hegemonia da heterossexualidade
	Formulários e sistemas de informações inadequados para casais homossexuais
	Discriminação de mães ou pais não biológicos
	Polêmicas sobre o fato de lésbicas serem mães
	A não redução da parentalidade a laços sanguíneos
Revelação da orientação sexual^19,21–25,31,34,38,41,43,47–50, 51,52,60,64,65,69,71^	Posicionamentos de casais sobre a não revelação porque não consideram isso importante
	Experiências negativas de casais por conta da revelação
	Defesa da revelação por parte de casais porque pode trazer uma atenção específica e demarcar um status a ser reconhecido
	Profissionais da medicina são menos propensos do que os da enfermagem a considerar que deve haver a revelação
Fertilização ^18^ ^,^ ^20^ ^,^ ^25^ ^,^ ^29^ ^,^ ^34^ ^,^ ^35^ ^,^ ^36^ ^,^ ^45^ ^,^ ^47^ ^,^ ^52^ ^,^ ^56^ ^,^ ^58^ ^,^ ^59^ ^,^ ^62^ ^,^ ^70^	Aspectos legais
	Dificuldade de acesso por parte de casais homossexuais
	Métodos
	Pares masculinos levam mais desvantagens do que pares femininos
	A legalidade da barriga de aluguel varia nos diferentes países
	Protocolos sobre reprodução assistida não atendem a casais do mesmo sexo
	Regulamento da fertilização in vitro dificulta o acesso de lésbicas
	Prioridade para as técnicas de inseminação do doador
	Proibição de gays doarem esperma
	Problemas legais no futuro com o doador
	Medidas para serviços de fertilização para casais homossexuais
Percepções de homossexuais e de profissionais sobre cuidados prestados e serviços de saúde em geral^16–19,22,24,25,27,28,30,31–34,37,38,41,44,46,49,50,53–55,59,61,63,64, 66–69,71,72^	Cuidados satisfatórios
	Cuidados insatisfatórios
	Interações negativas
	Ausência de apoio emocional
	Não aceitação de mães não biológicas
	Comentários homofóbicos
	Constrangimentos e discriminação
	Falta de benefícios para famílias chefiadas por um ou dois adultos homossexuais
	Abordagem exclusiva para o pai biológico
	Atendimento negado
	Curiosidade excessiva de profissionais
	Violência simbólica
	Hábitos dos serviços perturbados pela presença de casais homossexuais
	Linguagem inadequada dirigida a homossexuais
	Necessidade de haver ambiente para se proteger da homofobia
	Incompreensão por parte de profissionais
	Profissionais não preparados para atenção a casais homossexuais
Informações e ações educativas ^27^ ^,^ ^32^ ^,^ ^39^ ^,^ ^41^ ^,^ ^51^ ^,^ ^71^	Pontuação positiva para programas de educação sexual
	Informações insuficientes sobre lactação induzida para mães não biológicas
	Informações de cunho exclusivamente heterossexual
	Inscrição negada em grupo educativo

Os dados extraídos dos estudos foram agrupados em cinco temáticas, apresentadas em conjunto com seus respectivos subtemas no Quadro 3 . Percepções de homossexuais e de profissionais sobre cuidados prestados e serviços de saúde em geral foi a temática abordada por maior número de estudos (n = 31), seguida de contexto heteronormativo dos serviços de saúde (n = 26); revelação da orientação sexual (n = 20); fertilização (n = 14); e informações e ações educativas (n = 5).

## DISCUSSÃO

A produção científica da área da saúde em geral acerca da homoparentalidade afigura-se como uma questão cuja abordagem exige a compreensão de aspectos socioestruturais que ultrapassam esse campo do conhecimento. Pelo menos dois desses aspectos podem ser destacados. O primeiro deles diz respeito à heteronormatividade que, de uma forma hegemônica, faz com que – consciente ou inconscientemente – a primeira referência que se tem de família ou de parentalidade envolva a união de um homem cis com uma mulher cis. A existência de um casal homossexual faz com que essa norma heterossexual seja ou reafirmada para desqualificar tal casal ou desconstruída para se aceitar uniões e progenitores homoafetivos. Nesse sentido, constata-se que grande parte da literatura revisada, antes de tratar dos objetos específicos relacionados à homoparentalidade, menciona o contexto heteronormativo tanto como modelo explicativo para a não existência de ações de saúde específicas para casais de lésbicas ou de gays quanto como dimensão a ser questionada ou relativizada como referência única para se reivindicar uma atenção diferenciada voltada para esses casais.

Outro aspecto que emerge na produção científica revisada, que abrange questões que ultrapassam a área da saúde no trato da homoparentalidade, refere-se à legislação, ou ausência dela, que assegura ou veta não só a união de pessoas do mesmo sexo como também o desejo dessas pessoas de ter filhos. Tais aspectos, direta ou indiretamente, associam-se ao contexto heteronormativo. Observamos que, no que se refere aos aspectos legais, há uma variabilidade muito grande entre países e até mesmo no interior de estados que compõem um país. A ausência de dispositivos legais, sua incompletude deles e/ou dubiedade refletem diretamente na forma com os casais são atendidos ou não conseguem o acesso ao atendimento.

Revelar a orientação sexual, tanto na perspectiva de casais homossexuais quanto na de profissionais da saúde, emerge na literatura como algo polêmico. De um lado, a revelação pode contribuir para que haja uma especificidade nas ações de saúde voltadas para tais casais. Por outro lado, segundo alguns estudos, na percepção de lésbicas e gays, a revelação pode ter como consequências discriminações, questionamentos invasivos, preconceitos e até mesmo violência simbólica. O medo de revelar a homossexualidade, de certa forma, pode se articular com o contexto heteronormativo e as questões legais.

A produção científica sobre fertilização envolve questões relacionadas a legislação, direitos, dificuldades de acesso, ausência ou insuficiência de informações, exclusão de mães ou pais homossexuais não biológicos, pré-natal, parto, pós-parto e métodos. A literatura que trata desta temática se volta principalmente para lésbicas. No balanço feito nos resultados dos estudos, predominam as dificuldades de acesso à tecnologia de fertilização.

As percepções acerca da atenção à homoparentalidade, tanto por parte de casais homossexuais quanto de profissionais de saúde, em geral vinculam-se à existência de insatisfação frente aos cuidados recebidos e a atitudes negativas por parte de quem deveria prestar cuidados adequados.

No que se refere a informações e ações educativas, a literatura registra algumas experiências positivas. No entanto, concorre com essas experiências a percepção de que as informações são insuficientes. Ainda em termos educacionais, observa-se uma questão que atravessa todas as temáticas identificadas, de forma explícita ou implícita aos resultados: o despreparo dos profissionais de saúde para lidar não só com a homoparentalidade, mas também com a homossexualidade.

O acervo revisado constitui-se num mosaico de temas que, direta ou indiretamente, relacionam-se à homoparentalidade. Cada um deles, seja pelo que explicita seja por inferência do que está implícito, pode subsidiar princípios para o campo da saúde coletiva. Nesse sentido, os resultados desta revisão têm sua importância, uma vez que trazem subsídios para, dentre outros aspectos, a organização dos serviços de saúde, a implementação de ações específicas no âmbito da promoção da saúde da família e a formação adequada dos profissionais para abordar famílias de gays e lésbicas.

Observa-se ainda que o mapeamento obtido acerca do escopo do estudo é um ponto de partida para se ampliar a discussão acerca da temática central. Essa ampliação poderá ser mais exitosa na medida em que, ancorada nas referências socioantropológicas, possa problematizar questões voltadas para os diferentes arranjos familiares e outras concepções de parentescos que não se limitam à consanguinidade.

Por fim, destaca-se que, apesar do vasto acervo identificado, uma limitação que pode ser apontada para esta revisão é a filtragem de idioma, elegendo apenas as fontes em línguas portuguesa, espanhola e inglesa. Destaca-se, principalmente, a ausência de estudos na língua francesa, a qual deu origem ao termo homoparentalidade. Junto a isso, as próprias bases escolhidas para a pesquisa também podem ter influenciado no sentido de não haver estudos em francês.

## CONCLUSÕES

Dentre as principais conclusões, destaca-se que, embora a literatura nacional localizada acerca da homoparentalidade na área da saúde ainda seja tímida, a discussão internacional parece relativamente em ampla expansão. Em termos de evidências, podemos ressaltar que o escopo desta revisão não é problematizado de forma suficiente na formação e na atuação de profissionais de saúde; e os estudos quantitativos são inferiores em número, comparados aos de natureza qualitativa. Isso, ainda que nos traga as especificidades do tema central, não nos permite perceber a extensão da problemática apontada na maioria dos estudos.

O mapeamento da literatura acerca do assunto também revelou algumas lacunas na produção científica revisada. No âmbito da saúde coletiva, vale a pena ressaltar a insuficiência de estudos voltados para políticas e programas e a ausência de discussões sobre a saúde de crianças e adolescentes de famílias homoparentais.
